# Slitrk1 is localized to excitatory synapses and promotes their development

**DOI:** 10.1038/srep27343

**Published:** 2016-06-07

**Authors:** François Beaubien, Reesha Raja, Timothy E. Kennedy, Alyson E. Fournier, Jean-François Cloutier

**Affiliations:** 1 Montreal Neurological Institute, Centre for Neuronal Survival, 3801 University, Montréal, Québec, H3A 2B4, Canada; 2 Integrated Program in Neuroscience, McGill University, Canada; 3 Department of Neurology and Neurosurgery, McGill University, Canada

## Abstract

Following the migration of the axonal growth cone to its target area, the initial axo-dendritic contact needs to be transformed into a functional synapse. This multi-step process relies on overlapping but distinct combinations of molecules that confer synaptic identity. Slitrk molecules are transmembrane proteins that are highly expressed in the central nervous system. We found that two members of the Slitrk family, Slitrk1 and Slitrk2, can regulate synapse formation between hippocampal neurons. Slitrk1 is enriched in postsynaptic fractions and is localized to excitatory synapses. Overexpression of Slitrk1 and Slitrk2 in hippocampal neurons increased the number of synaptic contacts on these neurons. Furthermore, decreased expression of Slitrk1 in hippocampal neurons led to a reduction in the number of excitatory, but not inhibitory, synapses formed in hippocampal neuron cultures. In addition, we demonstrate that different leucine rich repeat domains of the extracellular region of Slitrk1 are necessary to mediate interactions with Slitrk binding partners of the LAR receptor protein tyrosine phosphatase family, and to promote dimerization of Slitrk1. Altogether, our results demonstrate that Slitrk family proteins regulate synapse formation.

One of the key steps in the development of the nervous system is the formation of new connections between different neurons. This process, referred to as synaptogenesis, also plays a critical role in the mature brain where the dynamic modification of circuitry has a profound effect on functions such as learning and memory. Multiple families of cell adhesion molecules have been implicated in various aspects of synapse formation, such as the formation of initial contacts and synapse maturation. Members of the neuroligin^1^,^2^, neurexin^1^,^3^, LRRTM^4^,^5^,^6^, SynCAM^7^,^8^, netrin G-ligand (NGL)^9^, SALM^10^, and EphB^11^ families of cell surface proteins are examples of such molecules involved in these processes.

It has been suggested that defects in neural connectivity or synaptic patterning underlie many neurodevelopmental disorders including autism and schizophrenia^12^. For example, familial forms of autism-spectrum disorders have been linked to mutations in neuroligin and neurexin (reviewed by Bourgeron^13^) as well as in SynCAM and cadherin^14^,^15^. Another family of transmembrane proteins that has been implicated in the etiology of brain disorders is the Slitrks. *SLITRK1* was proposed as a susceptibility gene for Gilles de la Tourette Syndrome^16^,^17^,^18^ and for the OCD spectrum disorder trichotillomania^19^,^20^, while variants of the *SLITRK2* gene have been found in patients with schizophrenia^21^. Mutations in Slitrk6 have been associated with myopia and deafness, and Slitrk family members may also associate with bipolar disorder^22^,^23^,^24^.

The Slitrks form a family of six structurally similar proteins that contain two leucine-rich repeat (LRR) domains in their extracellular portion and a cytoplasmic region that varies in size between members of the family^25^. LRR domains are protein-protein interaction regions commonly found in synaptogenic proteins^26^. Despite some overlap in their expression, the Slitrks display mostly distinct patterns of expression in the developing murine nervous system suggesting they may play specific roles in different regions of the brain^27^. In keeping with this possibility, gene ablation studies in mice for different Slitrk family members have yielded distinct phenotypes. While ablation of *Slitrk1* leads to increased anxiety-like behaviour^28^, *Slitrk5* mutant mice display obsessive compulsive-like behaviors^29^, and *Slitrk3* mutant mice exhibit increased susceptibility to seizures^30^. In contrast, *Slitrk6*-knockout mice display specific defects in development of the inner ear, including disorganized innervation and neuronal loss^31^. Based on the structure of Slitrks and the nature of phenotypes observed in some *Slitrk* mutant mice, Slitrks were proposed to regulate synapse formation in the central nervous system. Recent evidence has shown that Slitrk3 is specifically required for the formation of inhibitory synapses both *in vitro* and *in vivo*, and that other members of the Slitrk family can promote excitatory synapse formation^4^,^30^,^32^.

Here we have examined the function of two members of the Slitrk family, Slitrk1 and Slitrk2, in synapse formation. We show that Slitrk1 is preferentially localized to excitatory synapses. Overexpression of Slitrk1 or Slitrk2 can promote the formation of both excitatory and inhibitory synapses in cultures of hippocampal neurons. However, inhibition of Slitrk1 expression reduces the number of excitatory, but not inhibitory, synapses formed between hippocampal neurons in culture. We also demonstrate that the first LRR domain of Slitrk1 mediates interactions with the receptor tyrosine phosphatase PTP-δ, while the second LRR domain is necessary for dimerization of Slitrk1 at the cell surface. Taken together, our results demonstrate that Slitrk1 and Slitrk2 contribute to synapse formation and suggest that dimerization of Slitrk family members could be implicated in this process.

## Results

### Slitrk1 is preferentially localized at excitatory synapses

**Figure 1:**
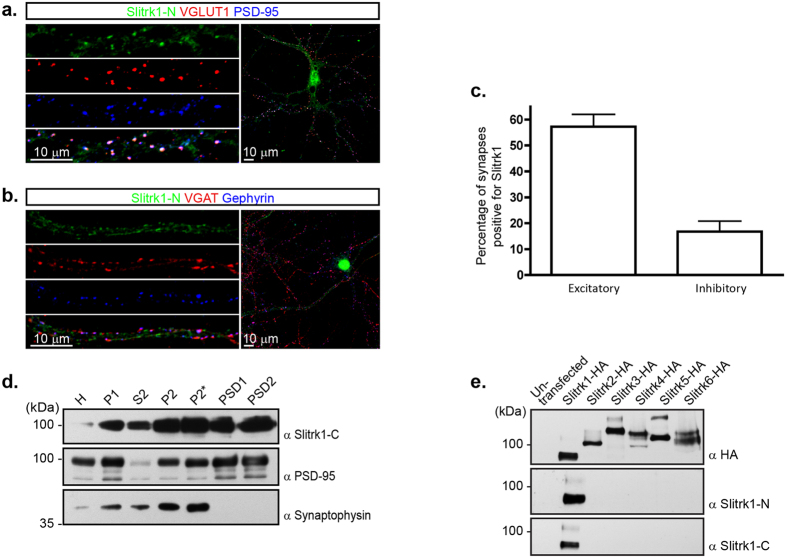
Localization of Slitrk1 at the synapse. (**a**,**b**) Subcellular localization of Slitrk1 in dissociated hippocampal neurons. Neurons at DIV15 were fixed and stained for Slitrk1-N (N-terminal antibody, green) with pre- (red) and post-synaptic (blue) excitatory (**a**) or inhibitory (**b**) markers. Slitrk1 staining is punctate and colocalizes mostly with excitatory synaptic puncta, as well as some inhibitory synaptic puncta. (**c**) Quantification of average number of Slitrk1-positive synapses per neuron. Mean ± s.e.m.: Excitatory, 57.23 ± 4.76, *n* = 6 neurons; Inhibitory, 16.74 ± 4.09, *n* = 5 neurons. Endogenous Slitrk1 localizes mainly to excitatory synapses in cultured hippocampal neurons. (**d**) Distribution of Slitrk1 in subcellular fractions of hippocampi isolated from 3–4 week old mouse brain. Note that Slitrk1 is detected in synaptic fractions including P2, P2* and the postsynaptic densities (PSD). PSD-95 and synaptophysin were probed for comparison. H, homogenate; P1, crude nuclear fraction; S2, supernatant after P2 precipitation; P2, crude synaptosomes; P2*, purified synaptosomes; PSD1, pellet after the first Triton X-100 extraction; PSD2, pellet after the second Triton X-100 extraction. (**e**) Characterization of Slitrk1 antibodies. Lysates of HEK293T cells expressing HA-tagged Slitrk family proteins were immunoblotted with Slitrk1-C (C-terminal antibody), Slitrk1-N, and HA antibodies. Slitrk1 antibodies specifically recognize Slitrk1.

The Slitrks are predominantly expressed in neural tissues at embryonic ages and postnatally^25^,^27^,^33^. More specifically, their localization at the synapse has been recently suggested based on the presence of tagged recombinant versions of these proteins at synaptic sites in cultured neurons. While Slitrk3 was localized to inhibitory synapses, other members of the Slitrk family appear localized to excitatory synapses^29^,^30^,^32^. To examine whether endogenous Slitrk1 is found at excitatory or inhibitory synapses, dissociated hippocampal neuron cultures were immunostained with an antibody against the Slitrk1 extracellular N-terminal region (Slitrk1-N) that does not recognize other Slitrk family members (Fig. 1a,b,e). In confocal images of these neurons, Slitrk1 signal was detected in a punctate pattern on hippocampal processes with a majority of these puncta colocalizing with VGLUT1- and PSD-95-positive excitatory synapses (Fig. 1a). A large proportion of VGLUT1- and PSD-95-positive clusters (57.23 ± 4.76%) were apposed to Slitrk1-positive puncta, while only 16.74 ± 4.09% of VGAT- and Gephyrin-positive clusters overlappedwith Slitrk1 puncta (Fig.1a–c). To further characterize the localization of Slitrk1 at the synapse, we examined its distribution in subcellular fractions generated from 3–4 week old mouse hippocampi using a specific antibody against the intracellular domain of Slitrk1 (Slitrk1-C). These analyses revealed that a portion of Slitrk1 protein is found in synaptic membrane fractions (Fig. 1d). As previously described, expression of Slitrk1 in an heterologous cell system leads to the detection of two closely migrating bands that likely represent differentially glycosylated forms of Slitrk1 (Fig. 1e)^34^. The Slitrk1-C and Slitrk1-N antibodies we have generated detect both forms of Slitrk1 proteins, but not other members of the Slitrk family (Fig. 1e). Taken together, our results indicate that Slitrk1 is localized to synapses and may thus play a role in synaptogenesis.

### Overexpression of Slitrk1 and Slitrk2 in neurons increases synapse density

**Figure 2:**
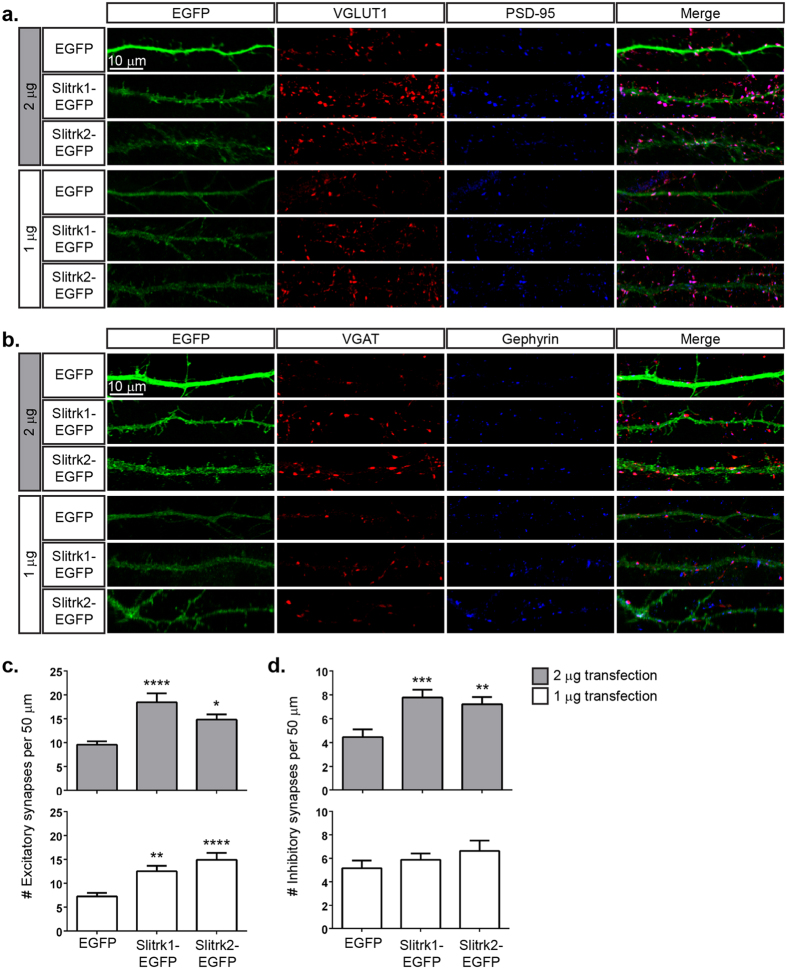
Overexpression of Slitrk1 and Slitrk2 in cultured neurons increases the amount of presynaptic excitatory and inhibitory contacts. (**a**,**b**) Cultured hippocampal neurons were transfected with either 1 or 2 μg of EGFP alone, Slitrk1 EGFP, or Slitrk2 EGFP at 13 DIV and immunostained for VGLUT1/PSD-95 (**a**) or VGAT/Gephyrin (**b**) at 15 DIV. (**c**,**d**) Quantification of the results from (**a**,**b**) respectively. (**c**) Average number of excitatory synapses per 50 μm, for 2 μg and 1 μg transfections Mean ± s.e.m. (Vector EGFP 2 μg, 9.58 ± 0.710, *n* = 36; Slitrk1 EGFP 2 μg, 18.47 ± 1.859, *n* = 36; Slitrk2 EGFP 2 μg, 14.83 ± 1.069, *n* = 36; From 3 separate experiments; ****p < 0.0001 *p < 0.05, One-way ANOVA and Vector EGFP 1 μg, 7.26 ± 0.745, *n* = 23; Slitrk1 EGFP 1 μg, 12.52 ± 1.142, *n* = 23; Slitrk2 EGFP 1 μg, 14.92 ± 1.473, *n* = 24; From 2 separate experiments; **p < 0.01 ****p < 0.0001 One-way ANOVA). (**d**) Average number of inhibitory synapses per 50 μm, for 2 μg and 1 μg transfections Mean ± s.e.m. (Vector EGFP 2 μg, 4.47 ± 0.642, *n* = 36; Slitrk1 EGFP 2 μg, 7.78 ± 0.649, *n* = 36; Slitrk2 EGFP 2 μg, 7.22 ± 0.600, *n* = 36; From 3 separate experiments; ***p < 0.001 **p < 0.01 One-way ANOVA and Vector EGFP 1 μg, 5.171 ± 0.646, *n* = 35; Slitrk1 EGFP 1 μg, 5.889 ± 0.533, *n* = 36; Slitrk2 EGFP 1 μg, 6.64 ± 0.876, *n* = 36; From 3 separate experiments; ns, *p* > 0.05 One-way ANOVA).

To determine whether Slitrk1 and another Slitrk family member, Slitrk2, can promote synaptic differentiation in neurons, we overexpressed Slitrk1 or Slitrk2 in hippocampal neurons. Cultured hippocampal neurons were transfected at 13 DIV with two different amounts of vector DNA (1 μg or 2 μg) expressing either EGFP, or EGFP-tagged Slitrk1 or Slitrk2. Cultures were immunostained two days later with antibodies against markers of glutamatergic excitatory synapses, VGLUT1 and PSD-95, or against markers of inhibitory GABAergic synapses, VGAT and Gephyrin, and the number of synapses was quantified. In agreement with previously published observations^32^, overexpression of either Slitrk1 or Slitrk2 induced a robust increase in excitatory synaptic differentiation in contacting axons, as measured by the number of VGLUT1-PSD-95 clusters (Fig. 2a,c). In contrast to previous observations where overexpression of Slitrk1 and Slitrk2 did not have an effect on inhibitory presynaptic differentiation^32^, we observed a significant increase in the number of inhibitory synaptic contacts as measured by the number of VGAT-Gephyrin-positive clusters (Fig. 2b,d, grey bars) on hippocampal dendrites. However, this effect appeared to be dependent on high levels of overexpression of Slitrk1 or Slitrk2 as transfecting these neurons with smaller amounts EGFP-tagged Slitrk1- or Slitrk2-expressing vectors led to increased numbers of excitatory, but not inhibitory, synapses (Fig. 2b,d, white bars). Hence, overexpression of either Slitrk1 or Slitrk2 can promote the formation of excitatory synapses in dissociated hippocampal cultures. Furthermore, they are capable of promoting inhibitory synapse formation when expressed at high enough levels.

### Knockdown of Slitrk1 reduces synapse number in hippocampal neuron cultures

**Figure 3:**
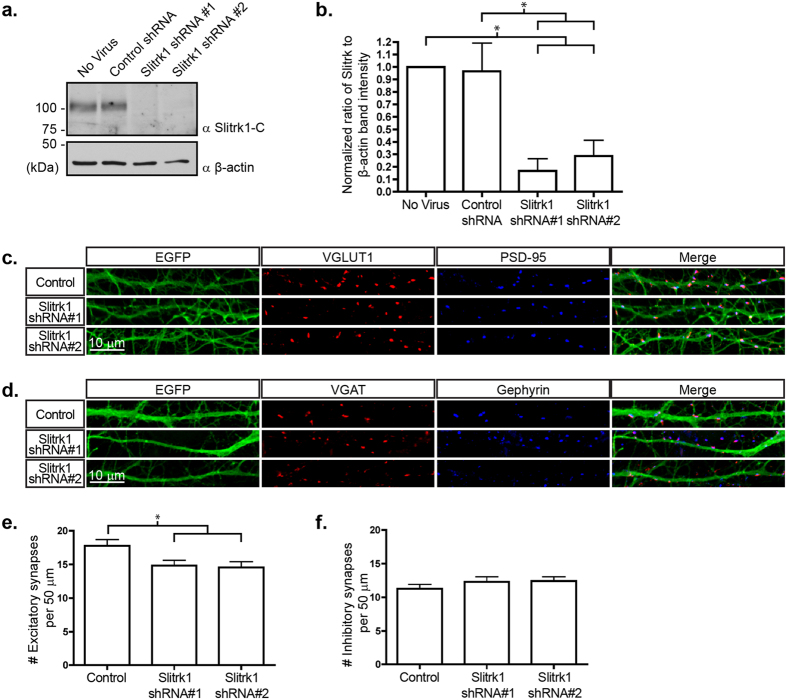
Knockdown of Slitrk1 by shRNA in cultured neurons reduces synapse density. Hippocampal neurons in culture were infected at 3 DIV with Slitrk1-shRNA lentivirus. At 10–11 DIV, cell lysates were immunoblotted for Slitrk1 to test knockdown. (**a**) Western blot analysis of Slitrk1 knockdown efficiency by shRNA #1 and #2. (**b**) Quantification of knockdown efficiency. Ratio of Slitrk1 to β-Actin band intensity normalized to untreated cells. Mean ± s.e.m. (Untreated, 1 ± 0.00; Control shRNA, 0.966 ± 0.226; Slitrk1 shRNA #1, 0.167 ± 0.098; Slitrk1 shRNA #2, 0.285 ± 0.128; From 3 separate experiments; *p < 0.05, One-way ANOVA). (**c**,**d**) Cultured hippocampal neurons were infected with Control, Slitrk1 shRNA #1 or Slitrk1 shRNA #2 at 3 DIV and immunostained at 10–11 DIV for VGLUT and PSD-95 (**c**) or VGAT and Gephyrin (**d**) to label excitatory or inhibitory synaptic puncta, respectively. (**e**) Average number of excitatory synapses per 50 μm, Mean ± s.e.m. (Control siRNA, 17.76 ± 0.96 *n* = 75; Slitrk1 siRNA #1, 14.84 ± 0.781 *n* = 80; Slitrk1 siRNA #2, 14.56 ± 0.857 *n* = 73; From 5 separate experiments; *p < 0.05, One-way ANOVA). (**f** ) Average number of inhibitory synapses per 50 μm, Mean ± s.e.m. (Control siRNA, 11.28 ± 0.649 *n* = 47; Slitrk1 siRNA #1, 12.31 ± 0.760 *n* = 36; Slitrk1 siRNA #2, 12.42 ± 0.629 *n* = 33; Done in 3 separate experiments; One-way ANOVA).

To test whether endogenous Slitrk1 is required for excitatory or inhibitory synapse formation, we used RNA interference to knock down expression of Slitrk1 in hippocampal neurons. We generated two lentiviral vectors expressing individual shRNAs targeting Slitrk1 and infected dissociated hippocampal neuron cultures. These two independent shRNA constructs reduced the expression of endogenous Slitrk1 by 82.7% and 70.5% in hippocampal neurons (Fig. 3a,b). Knockdown of Slitrk1 significantly reduced the number of VGLUT1- and PSD-95-positive excitatory synapses (Fig. 3c,e) but did not affect the number of VGAT- and Gephyrin-positive inhibitory synapses (Fig. 3d,f), indicating that Slitrk1 specifically contributes to excitatory synapse differentiation.

### The second leucine-rich repeat of Slitrk1 is necessary for its homophilic interaction at the cell surface

**Figure 4:**
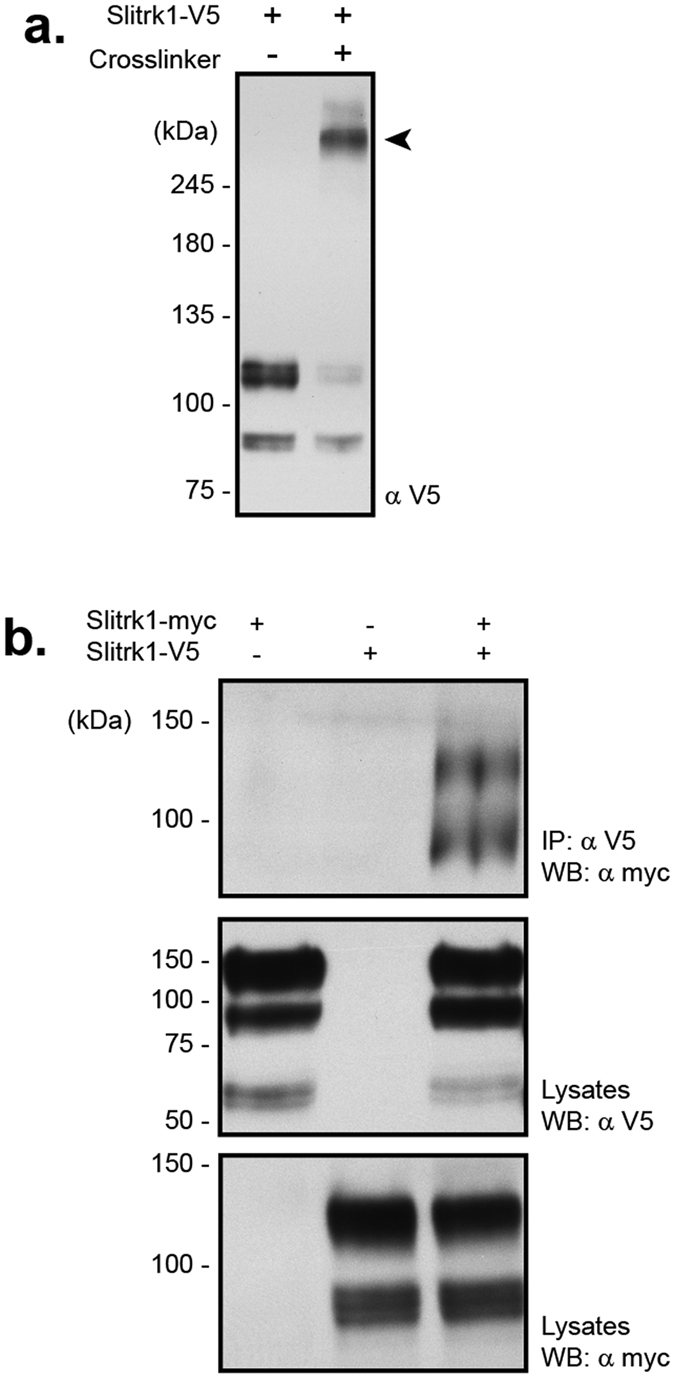
Slitrk1 molecules have the ability to interact at the cell surface. (**a**) Slitrk1 exists in a complex at the cell surface. Western blot of cross-linked (+) or mock-treated (−) HEK293T cells transfected with Slitrk1-V5. Cross-linked sample reveals a protein complex containing Slitrk1-V5 that migrates above 245 kDa (arrowhead). (**b**) Slitrk1 forms homophilic complexes. HEK293T cells were transfected with either Slitrk1-V5 alone, Slitrk1-myc alone, or co-transfected with both tagged vectors. Protein lysates were immunoprecipitated with V5 antibodies and western blots were performed using myc and V5 antibodies. These results indicate that Slitrk1 can form homophlic complexes when expressed in HEK293T cells. IP, Immunoprecipitation; WB, Western Blot.

The extracellular region of Slitrk1 contains two leucine-rich repeats, which are domains that can mediate protein-protein interactions and dimerization of cell surface receptors^35^,^36^. To examine whether Slitrk1 molecules can interact in *cis* at the cell surface, we used a chemical cross-linking approach to promote the maintenance of *cis*-interacting complexes at the plasma membrane. V5-tagged Slitrk1 was expressed in HEK293T cells and native plasma membrane complexes were preserved through covalent cross-linking. The SDS-PAGE migration of Slitrk1 corresponded to two bands at molecular weights of approximately 85 and 120 kDa. While the band migrating at 120 kDa represents the mature form of Slitrk1 expressed at the surface (Fig. 4a), the lower molecular weight band likely represents an immature form of Slitrk1^34^. After cross-linking, the majority of Slitrk1 immunoreactivity appeared as a single band corresponding to an approximate molecular weight of 260 kDa, suggesting that it may be composed of Slitrk1 homodimers (Fig. 4a, arrowhead). Furthermore, V5- and Myc-tagged Slitrk1 molecules can be co-immunoprecipitated from lysates of HEK293T cells expressing these two proteins (Fig. 4b). Taken together, these two results indicate that Slitrk1 molecules have the ability to interact with each other at the cell surface, possibly forming homodimers.

**Figure 5:**
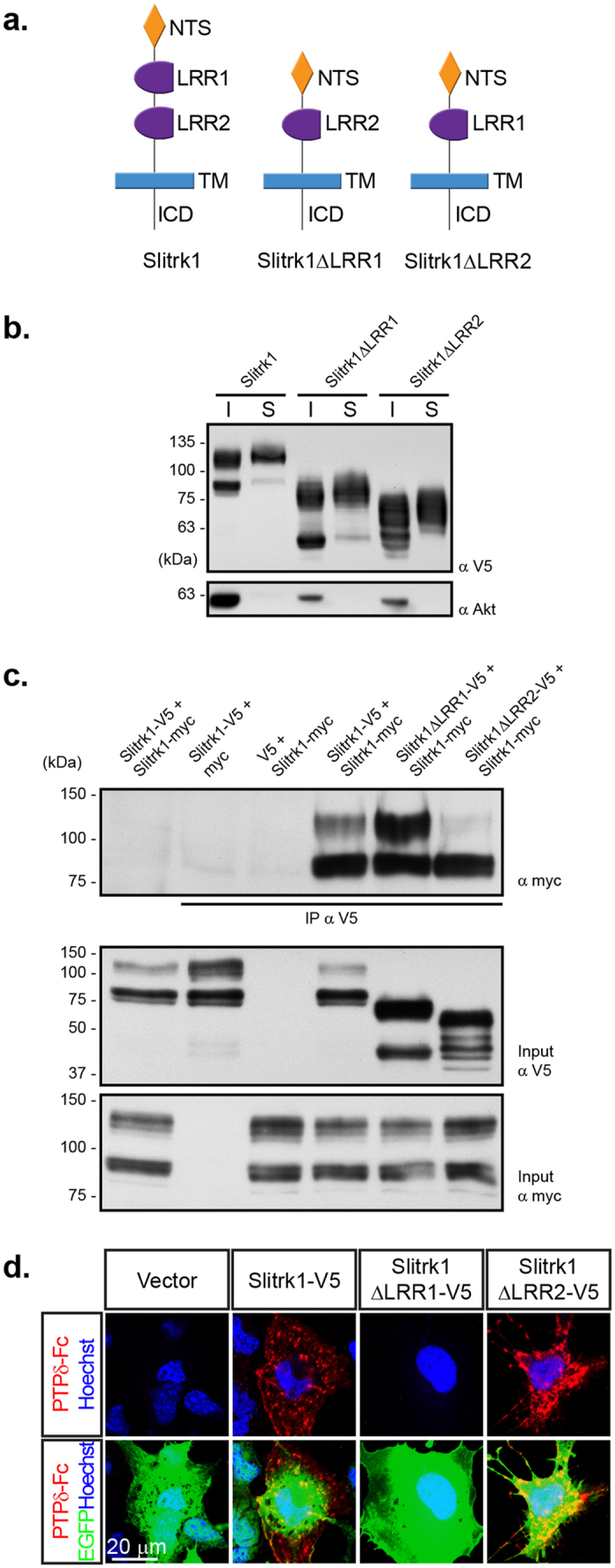
Determination of the LRR domain required for homophilic binding and binding to PTPδ. (**a**) Diagram of Slitrk1 mutant constructs. Mutants, Slitrk1ΔLRR1 and SlitrkΔLRR2, are missing the coding region for leucine-rich repeat domain 1 and 2, respectively. Full length and mutant constructs are C-terminally V5-tagged. NTS, n-terminal sequence; LRR, Leucine-rich repeat domain; TM, Transmembrane domain; ICD, intracellular domain. (**b**) Expression of full length and mutant V5-tagged Slitrk1 protein at the cell surface. COS cells were transfected with Slitrk1-V5 or mutant variants. Cell-surface proteins were isolated by incubating cells with biotin, followed by immunoprecipitation with avidin-conjugated beads. Biotinylated cell surface Slitrk1-V5 was detected by blotting with a V5 antibody. The intracellular protein Akt was not detected in these immunoprecipitates. Expression of the Slitrk1 mutants at the cell surface is similar to that of full length Slitrk1. I, Input; S, Surface protein. (**c**) Analysis of interaction between Slitrk1 full length and mutant constructs by co-immunoprecipitation. HEK293T cells were either transfected with Slitrk1-myc or Slitrk1-V5 alone, or co-transfected with Slitrk1-myc and one of the Slitrk1-V5 full length or mutant constructs. Lysates were immunoprecipitated with V5 antibodies and blotted with myc and V5 antibodies. These results indicate that the LRR2 domain of Slitrk1 is required for homophilic Slitrk1 interactions. (**d**) Cell surface binding assay performed on COS cells transfected with V5-tagged Slitrk1 variants. Cells were treated with purified PTPδ-Fc protein and analyzed by immunofluorescence for PTPδ-Fc binding (red) and V5 (green). These results demonstrate that the LRR1 is required for the binding of PTPδ to Slitrk1.

Since leucine-rich repeats have been implicated in the dimerization of cell surface receptors, we examined the requirement of the two LRR domains of Slitrk1 for this interaction. V5-tagged Slitrk1 molecules containing deletions of either the first (Slitrk1ΔLRR1) or second (Slitrk1ΔLRR2) LRR domains (Fig. 5a) were expressed in HEK293T cells with a Myc-tagged full-length Slitrk1 to perform co-immunoprecipitation experiments. Both Slitrk1 deletion proteins were expressed at the cell surface at similar levels to the wild-type Slitrk1 (Fig. 5b). While deletion of the first LRR domain did not affect the co-immunoprecipitation of this mutant with Slitrk1, we observed a robust decrease in the interaction of Slitrk1ΔLRR2 with Slitrk1, indicating that the second LRR contributes to Slitrk1-Slitrk1 interactions (Fig. 5c). Interestingly, removal of the second LRR did not affect the binding of Slitrk1 to PTPδ in a cell binding assay, indicating that improper folding of this deletion mutant is unlikely to account for the lack of interaction with full length Slitrk1 (Fig. 5d). In contrast, removal of the first LRR prevented Slitrk1 binding to PTPδ, confirming this previously published observation (Fig. 5d)^32^. Taken together, these results demonstrate that Slitrk1 can form homodimers at the cell surface and that the LRR2 domain is necessary for this interaction to take place.

## Discussion

Members of the Slitrk family of proteins have been implicated in the etiology of multiple neuropsychiatric disorders^16^,^17^,^18^,^19^,^20^,^21^,^23^,^24^. Here, we show that Slitrk1 is present in postsynaptic density fractions isolated from mouse hippocampi and that Slitrk1 is preferentially localized to excitatory synapses in hippocampal neurons, supporting a role for these proteins in regulating synapse formation. We also demonstrate that overexpression of Slitrk1 or Slitrk2 in hippocampal neurons promotes the formation of both excitatory and inhibitory synapses, which is consistent with the previously reported observation that Slitrk1 can promote presynaptic clustering of both VGLUT1 and VGAT in a mixed-culture assay^30^. However, considering that Slitrk1 is preferentially localized at excitatory synapses (Fig. 1a–c) and that its overexpression has been shown to specifically enhance the formation of excitatory synapses^32^, it is somewhat surprising that we also observe an increase in inhibitory synapse formation in our experiments. It is therefore likely that the enhanced number of inhibitory synapses we observe in these neuronal cultures results from high overexpression of Slitrk1 in our system leading to its ectopic localization to inhibitory synapses. In keeping with this possibility, transfecting hippocampal neurons with lower amounts of EGFP-tagged Slitrk1 vector promotes formation of excitatory synapses but not inhibitory synapses (Fig. 2). Decreased expression of Slitrk1 in hippocampal neurons reduced the number of excitatory, but not inhibitory, synapses formed, demonstrating that endogenous Slitrk1 specifically contributes to excitatory synapse formation (Fig. 3). Taken together, our observations and previously published results indicate that Slitrk1 has the potential to promote presynaptic differentiation of both excitatory and inhibitory synapses, but that its localization restricts its effect to excitatory synapses.

Slitrk3 has been identified as a specific inducer of inhibitory presynaptic differentiation by binding to the receptor protein tyrosine phosphatase, PTPδ, on the presynaptic side of the cleft^30^. Ablation of *Slitrk3* in mice leads to specific reductions in both inhibitory synapse density and synaptic transmission in the hippocampus^30^. In contrast to the specific role that Slitrk3 plays in regulating inhibitory synapse formation, other Slitrk family members have been shown to modulate excitatory synapse formation by interacting with PTPσ^32^. Our results demonstrate for the first time that endogenous Slitrk1 proteins are localized to excitatory synapses and confirm that Slitrk1 contributes to the formation of these synapses *in vitro*.

The presence of two LRR domains in the extracellular region of Slitrk molecules suggests that protein-protein interactions play a critical role in their functions. Indeed, the most N-terminal LRR domain (LRR1) of Slitrk1 is essential to mediate its interaction in *trans* with LAR-RPTPs and for its ability to promote synapse formation (Fig. 5d)^37^. Interestingly, this domain is also necessary for the interaction of another Slitrk family member, Slitrk5, with a cell surface receptor in *cis* to regulate its cell surface expression. The LRR1 domain of Slitrk5 was recently shown to mediate interactions with the receptor tyrosine kinase TrkB and to regulate its trafficking inside the cell. The binding of BDNF to TrkB promotes an interaction between Slitrk5 and TrkB, outcompeting PTPδ binding^38^. In contrast to the LRR1 domain, binding partners for the second LRR2 domain of Slitrks remain to be identified. Our observation that Slitrk1 molecules can form complexes at the cell surface through interactions between their LRR2 domains when expressed in HEK293T cells suggests that other Slitrk family members may also be capable of forming homodimers at the surface. While it remains unclear whether dimerization of Slitrk1 is required for its function at the synapse, dimerization and lateral interactions of other synaptogenic proteins have been implicated in their ability to promote synapse formation^39^,^40^. For example, the dimerization of neuroligin is necessary for its synaptogenic activity, and has been proposed to regulate the transsynaptic clustering of neurexin in the presynaptic terminal during synapse assembly^40^. While dimerization of Slitrk1 does not appear to be necessary for its binding to PTPδ (Fig. 5d), it may serve to promote the lateral assembly of LAR-RPTPs-Slitrk complexes that has been proposed to take place at the synapse^37^. Future studies should shed light on the role of Slitrk1 dimerization through LRR2 for its function at the synapse.

## Materials and Methods

### cDNA constructs

Full-length human *Slitrk1* (aa 1–696) and human *Slitrk2* (aa 1–845) were sub-cloned into the pEGFP-N1 vector (Clontech). For C-terminal V5- or MYC-tagged Slitrk1 constructs, full-length human *Slitrk1* (aa 1–696) was sub-cloned into the pcDNA3.1 MYC-His A vector and the pcDNA3.1 V5-His A vector (Invitrogen), respectively. Slitrk1 mutants lacking either of the LRR domains (*Slitrk1*Δ*LRR1* (Δ aa 18–264) and *Slitrk1*Δ*LRR2* (Δ aa 304–599)) were also each sub-cloned into the pcDNA3.1 V5-His A vector. For knockdown of Slitrk1 in hippocampal neurons, shRNA sequences targeting nucleotides against rat Slitrk1 (Target #1: nucleotides 1222–1242; Target #2: nucleotides 2214–2234) were cloned into pcDNA6.2/GW-EmGFP-miR plasmid (gifted by Dr. Peter S. McPherson). PTPδ-Fc and HA-tagged Slitrk1 to Slitrk6 constructs were gifted to us by Dr. Hideto Takahashi and Dr. Ann Marie Craig, respectively.

### Antibodies

A rabbit polyclonal antibody recognizing the intracellular portion of Slitrk1 (referred to as Slitrk1-C) was generated against the peptide DGSHRVYDCGSHS (aa 680–693 of the mouse sequence) and purified against the same epitope. Another Slitrk1 rabbit polyclonal antibody (namely Slitrk1-N) was obtained from an animal immunized with the complete extracellular portion of the protein (aa 2–600). The other antibodies were obtained commercially; Akt (rabbit, New England Biolabs); β-Actin (mouse, Abcam); Gephyrin (mouse, SynapticSystems); GFP (rabbit, Invitrogen); HA (mouse, Sigma); myc (goat, Abcam); PSD-95 (mouse, NeuroMab); Synaptophysin (Mouse, SynapticSystems); V5 (rabbit, Invitrogen); VGAT (guinea pig, SynapticSystems); VGLUT1 (guinea pig, SynapticSystems).

### Cell culture

All procedures involving the use of animals were approved by the animal care committee of the Montreal Neurological Institute and performed in accordance with the approved guidelines. E18-19 rat embryos were obtained from Sprague Dawley females (Charles River). Cultures of hippocampal neurons were prepared from the embryos according to previously described protocols^41^. Briefly, hippocampi were isolated from the embryos, Cells were trypsinized for 15–20 minutes in 0.25% Trypsin-EDTA (gibco, Life technologies), washed in Neurobasal media (Invitrogen, Life Technologies) supplemented with L-Glutamine (gibco, Life technologies), Pen/Strep (gibco, Life technologies), and B-27 (gibco, Life technologies), triturated using flamed glass pasteur pipettes, and then plated onto Poly-L-lysine-coated (Sigma) coverslips (Fisherbrand, 12CIR-1D) in 24-well plates and grown at 37 °C, 5% CO_2_.

### Slitrk1 knockdown

lentiviral shRNA vectors targeting Slitrk1 contained the following nucleotide sequences: Slitrk1shRNA#1, 5′-AGCACCCTACCTGCTAATGTA-3′; Slitrk1shRNA#2, 5′-TAAGCTCAGTCTGCACAATAA-3′. Lentiviral vectors and virus was produced according to Allaire *et al.*^42^. Oligonucleotide sequences targeting Slitrk1 were cloned into the lentiviral expression vector pRRLsinPPTeGFP. HEK293T cells were then transfected with this vector, together with pMD2.g and pRSV-Rev and pMDLg/pRRE which encode necessary viral genes. Media was collected at 24, 36, and 48 hrs post-transfection and concentrated by centrifugation. Hippocampal neurons were infected at 3 DIV with control or shRNA lentivirus and were either fixed for immunostaining or lysed at 10–11 DIV. Knockdown efficiency was verified by Western blotting of hippocampal neuron lysates with the Slitrk1-C antibody.

### Transfection and immunocytochemistry

HEK cells and cultured hippocampal neurons were transfected using Lipofectamine2000 Reagent (Invitrogen, Life Technologies). For HEK cell transfections, DNA to Lipofectamine2000 ratios were used as recommended by manufacturer’s guidelines. Cultured neurons were transfected at 13 DIV for 15 minutes with 1 or 2 μg DNA and 0.5 μL Lipfectamine2000 per well. Cells were fixed with 4% paraformaldehyde/4% sucrose (vol/vol) at 15 DIV, and permeabilized with 0.2% Triton X-100 (vol/vol) in phosphate-buffered saline (1 × PBS, pH = 7.4). All cells undergoing immunocytochemistry were blocked in 5% FBS diluted in 1 × PBS and then incubated with the appropriate primary antibodies overnight at 4 °C, followed by Alexa-488-, Alexa-546-, Alexa-647-conjugated species-specific secondary antibodies (1:400; Invitrogen). Coverslips were mounted onto microscope slides using Fluoromount-G (Southern Biotech).

### Image acquisition and quantification

Images were acquired using a confocal microscope with a 63× oil objective (LSM710; Zeiss). The settings were kept constant for all scanning in each experiment. The number of pre- and post-synaptic puncta were counted along 50 μm lengths of axons. All values are presented as mean ± s.e.m., and significance of the quantification was determined by ANOVA followed by Tukey’s multiple comparisons test.

### Production of PTPδ-Fc protein

Soluble PTPδ-Fc was purified as previously described^30^. HEK293T cells were first transfected with the PTPδ-Fc plasmid. Secreted protein was purified by affinity chromatography using protein G-sepharose 4 Fast Flow beads (GE Healthcare), eluted with 100 mM Glycine, pH 2.7 (immediately neutralized with 1 M Tris pH 9.0), and concentrated using Amicon Ultra centrifugal filter units (30 kDa cutoff, Millipore).

### Cell surface binding assay

COS cells were grown on Poly-L-Lysine-coated coverslips and transfected with Slitrk1-V5 full length or mutant variants (Slitrk1ΔLRR1-V5 and Slitrk1ΔLRR2-V5), or the V5 vector alone as a control. Binding was performed as previously described^30^. Cells were grown for 48 hours, then washed with extracellular solution (168 mM NaCL, 2.4 mM KCl, 20 mM HEPES pH7.4, 10 mM D-glucose, 2 mM CaCl_2_, 1.3 mM MgCL_2_) with 200 ug/mL BSA (ECS/BSA). Cells were then treated with purified PTPδ-Fc for 1 hour at room temperature and fixed for 12 minutes in 4% PFA in 1 × PBS. After blocking in 3% BSA, 5% FBS in 1 × PBS, cell were incubated with Alexa546-conjugated Donkey anti-Human IgG for one hour at room temperature, stained for nuclei using Hoechst 33342 (1:7500, Molecular probes) and mounted onto miscroscope slides using Fluoromount-G (Southern Biotech).

### Crosslinking

HEK293T cells were transfected with full length V5-tagged Slitrk1 plasmid. 48 hours post-transfection, cells were treated with 1.0 mM bis[sulfosuccinimidyl] suberate (BS^3^, Thermo Scientific) for 30 minutes at 4 °C. Cross-linker reaction was quenched with 15 mM Tris pH8.0 for 15 minutes at RT. Cells were then lysed in lysis buffer (1% SDS, 5 mM EDTA, 50 mM Tris, 150 mM NaCL), sonicated and run on an SDS-page gel for Western Blotting analysis using the V5 antibody.

### Synaptosomal fractionation

PSD fractionation was performed on hippocampi isolated from 3–4 week old mouse brain as described^43^ with some minor modifications: a purified P2 pellet was incubated in 0.5% Triton X-100, 40 mM Tris-HCl (pH = 8.0) and then centrifuged at 32,000 × *g* to generate the PSD1 fraction. This fraction was then further extracted with 0.5% Triton X-100, 40 mM Tris-HCl (pH = 8.0), and subsequently centrifuged at 200,000 × *g* to isolate the PSD2 fraction. Samples were subject to Western Blotting analysis.

### Cell surface biotinylation

COS cells were transfected with the different plasmids for 48 hours, washed 3 times with 1 × PBS, incubated with EZ-Link Sulfo-NHS-LC-Biotin 1 mg/ml (Thermo Scientific Pierce) at 4 °C for 30 min, and washed 3 times with 1 × PBS + 100 mM glycine to quench the biotin reagent. The cells were then lysed and surface proteins were isolated using streptavidin agarose beads (Thermo Scientific Pierce) prior to running samples on an SDS-page gel for Western Blotting analysis.

### Immunoprecipitation and Western Blotting

For immunoprecipitation, HEK293T cells were doubly transfected with Slitrk1-myc (full length Slitrk) as well as Slitrk1-V5 or V5-tagged Slitrk1 variants. 750 ug cell lysates were incubated with anti-V5 for 2 hours at 4 °C and then incubated with Protein A/G Plus Agarose beads (Santacruz) for 2 hours at 4 °C. Beads were then washed and immunoprecipitate was eluted using sample buffer and β-mercaptoethanol and heated for 10 minutes at 95 °C. Samples were run alongside initial protein lysate (input) on an SDS-page gel. Proteins were transferred onto PVDF membranes, blocked with 5% milk in 1 × TBST before probing with antibodies.

## Additional Information

**How to cite this article**: Beaubien, F. *et al.* Slitrk1 is localized to excitatory synapses and promotes their development. *Sci. Rep.* **6**, 27343; doi: 10.1038/srep27343 (2016).
